# Performance evaluation of a firewall service based on virtualized IncludeOS unikernels

**DOI:** 10.1038/s41598-024-51167-8

**Published:** 2024-01-04

**Authors:** Tytus Kurek, Marcin Niemiec, Artur Lason

**Affiliations:** AGH University of Krakow, Mickiewicza 30, 30-059 Kraków, Poland

**Keywords:** Engineering, Electrical and electronic engineering

## Abstract

Network function virtualization technology has long moved beyond the experimental phase to become a standard in the implementation of modern telecommunications networks. It is anticipated that in the near future all network services will be implemented in software based on cloud-native architecture. As a result, telecommunications service providers have started exploring containers and unikernels as alternative technologies to traditional virtual machines. This paper presents performance evaluation of a firewall service based on IncludeOS unikernels. It shows that IncludeOS unikernels achieve promising performance results compared to Ubuntu-based virtual machines and containers. The presented evaluation is based on a number of experiments and benchmarks performed to investigate how different parameters of a firewall service change depending on the number of firewall rules.

## Introduction

The growing demand for better user experience in mobile networks in recent years has forced network operators to modernize their infrastructure. This process, although long and expensive, has already begun. An inseparable part of this process is softwarization of network services for better economics and improved flexibility. Network Function Virtualization (*NFV*)^1^ is a technology allowing Telecommunications Service Providers (*TSPs*) to migrate their legacy hardware-based network services to software-based ones.

One of the main challenges in the implementation of NFV technology is ensuring the same performance of software-based network services as for their hardware-based equivalent^2^. First implementations of software-based network services used virtual machines (*VMs*) which affects the performance of the workloads. Although various performance extensions, such as Single-Root Input/Output Virtualization (*SR-IOV*) or Data Plane Development Kit (*DPDK*), aim to improve network performance of the workloads, they usually introduce an additional cost and complexity to the underlying infrastructure^3^. In response to the aforementioned challenges, TSPs have recently started exploring alternatives to traditional VMs.

An example of such a technology are containers. As containers do not rely on a hypervisor, their performance is close to the performance of applications running directly on a physical machine. Moreover, their fine-grained nature makes them suitable for the implementation of cloud-native network services based on the microservices architecture. As a result, containers are being explored by the telco world. Network functions implemented based on containers, referred to as Container Network Function (*CNFs*), are proposed in^4^. The ability to deploy CNFs on Kubernetes has also been recently announced by one of the leading open source Management and Orchestration (*MANO*) platforms, Open Source MANO (*OSM*)^5^.

However, security concerns are a major challenge of CNF implementation. Containers are known to be less secure than VMs^6^. This is because containers use a shared kernel, relying on the internal kernel’s features to provide an isolation of the workloads. While containers run as separate processes on the host’s kernel, VMs use a hypervisor to provide hardware virtualization. As a result, each Virtual Network Function (*VNF*) runs on its own kernel. As security considerations are just as important as performance when implementing software-based network services, this undermines containers as the target technology for NFV.

Another alternative to traditional VMs are unikernels^7^. Although these images run as VMs, they are smaller, faster and more lightweight than regular cloud images. This is due to the construct of unikernels which only includes the minimum set of kernel libraries required to run the application. Unikernels achieve much better performance results than VMs, as well as providing higher level of security than containers. This combination of performance and security makes unikernels promising candidates for the implementation of network services. Network functions based on unikernels, referred to as Unikernel Network Functions (*UNFs*), are recently proposed in^8^. The following paper expands on this work by providing further experimental results and extensive analysis. This aims to evaluate performance and confirm the usability of this novel solution.

The remainder of the paper proceeds as follows. Related work is reviewed in “Related work”. “From unikernels to unikernel network functions” provides an introduction to unikernels and UNFs by presenting network functions evolution and the rationale behind using unikernels in network services. “Performance evaluation” describes the method used to evaluate performance results of a firewall service based on IncludeOS unikernels. The extensive experimental results are presented and discussed in “Observations and discussion”. All observed limitations and directions for future work are documented in “Limitations and future work”. Finally, “Conclusions” concludes the paper.

## Related work

The authors of^9^ present their research into unikernel and containers performance in firewall applications. They examine basic performance metrics of a firewall service with a fixed number of rules, implemented in IncludeOS and Docker. They note that performance results of a unikernel-based firewall vary depending on the metric being examined. Similar findings are reported by Behravesh et al. in^10^ where they analyze basic performance metrics of the Apache and Redis services implemented in three different ways: as a VM running on the Kernel Virtual Machine (*KVM*) hypervisor, as a Docker container, and as a Rumprun unikernel.

In^11^ the authors attempt to compare the performance of VM, container and unikernel-based NFV solutions implemented with x86 and ARM architectures. A comparative analysis of this type could be promising in terms of VNF energy efficiency. The performance of selected virtualization scenarios (KVM, Docker, rkt, Rumprun and OSv) was evaluated and compared in terms of Central Processing Unit (*CPU*) and memory efficiency and network throughput. Unfortunately, in the case of unikernel-based scenarios, it was not possible to obtain any valuable test results in the ARM environment. The authors conclude that this is due to insufficient support of unikernel projects for 64-bit ARM architectures. The results of tests performed in the x86 architecture show a significant dependence of the performance on the specific implementation.

More research into unikernel performance is presented in^12^. The authors compare the performance of unikernel and container-based Representational State Transfer (*REST*) microservices implemented with three programming languages: Go, Java and Python. The Unikernel instances were built using the OSv platform, while the container test bed was set up with Docker tools. Tests of single thread applications show significantly better performance in microservices implemented in unikernels. The authors show that microservices written in Go perform with a 38% higher efficiency, while those implemented in Java and Python perform with a 16% higher efficiency. Test results for multithread applications are less impressive and significantly less clear. In multithread scenarios, containers show significant advantages, in particular in scenarios not involving intense context switching between user space and kernel. The author of^13^ provided a comparative performance assessment of firewall instances based on Ubuntu Linux and IncludeOS. The firewall instantiated on the IncludeOS platform was developed using the NaCl programming language, designed explicitly for the IncludeOS environment. However, test scenarios covered firewalls with limited number of rules: 10,000 and 50,000. The contribution of this paper significantly expands the research scope by investigating the implementation of a firewall with a significantly larger number of rules—up to one million rules. This extension in the scale of rule implementation represents a noteworthy trend in the increasing number of terminals served in evolving mobile networks and demonstrates its efficacy even under the demanding scenario of managing an extensive ruleset.

Security aspects of the unikernel approach to virtualization are discussed in detail in^14^. The paper presents valuable analysis of differences in potential attack vectors specific to VMs, containers and unikernels, as well as reporting in-depth analysis of potential unikernel vulnerabilities and methods for their mitigation. The authors also discuss differences between types and versions of unikernels, focusing on potential unikernel use cases.

The following paper is a continuation and expansion on^8^ in which we present an innovative concept of unikernel application to network function virtualization. Performance evaluation of network services implemented in various technologies and performance evaluation of unikernels themselves have been hot research topics in recent years. Our research on unikernel performance focuses on the efficiency of networking services, unikernel image size and launch time.

## From unikernels to unikernel network functions

Put simply, UNFs are network services implemented based on unikernels. In order to understand how UNFs work, it is useful to first examine the evolution of the NFV technology and take a closer look at unikernels themselves.

### NFV technology evolution

The NFV technology has been widely adopted by leading TSPs around the world in recent years^15^. The success of NFV is the result of the spectacular success of the underlying cloud computing technology, well-conducted standardization, and most of all—the measurable benefits that this technology brings to telcos. NFV lowers the Total Cost of Ownership (*TCO*) associated with maintaining telecommunications infrastructure. By migrating network services from legacy appliances to the cloud, TSPs achieve better resource utilization and accelerated software development. This drives innovation, brings down costs of the services provided, and improves competition on the market.

However, the evolution of NFV technology has not been straightforward. The original aim was simply to virtualize legacy, monolithic network services and run them entirely inside of VMs. While this approach has been partially successful, it is not scalable and causes problems in the long run, for example related to day-2 operations. As a result, TSPs began to redesign their network services based on the microservices architecture. Container technologies emerged at a similar time, providing improved performance and cloud-native architecture of network services. Thus, the interest of TSPs shifted from VMs to containers.

Due to their fine-grained nature, containers are better suited as building blocks for cloud-native network services. Moreover, because containers run directly on top of the kernel, bypassing the entire hardware virtualization layer, they achieve better performance results than traditional VMs. This makes them suitable for NFV use cases such as 5G core and virtual Evolved Packet Core (*vEPC*). However, containers also have several limitations. Due to the fact that containers use a shared kernel, it is impossible to achieve the same level of security and isolation in container-based network services as in those based on a hypervisor. As a result, TSPs often run separate container coordination platforms, such as Kubernetes, for each individual network service to achieve true multi-tenancy. This approach is also non-scalable and increases the TCO associated with NFV Infrastructure (*NFVI*) maintenance.

The introduction and extensive implementation efforts of 5G networks has drawn attention to the problems of network slicing. A network slice is defined as a logically separated end-to-end part of the 5G physical network including User and Control Plane Network Functions. In order to fulfill all slicing functional requirements it is necessary to virtualize, strictly isolate and efficiently secure networking resources and virtual machines running functions responsible for network control and operations. Virtualized end-user authorization for separated slices and operated by different tenants needs to be implemented using reliable, protected and cloud-optimized tools and solutions. The requirements are becoming increasingly more demanding as dynamic slices are considered^16^. Dynamic slicing promises more flexible allocation of physical network resources to individual slice instances, thus optimizing costs of network operation and improving perceived service quality.

Dynamic slicing can be implemented to dynamically create new slice instances and on-demand slice scaling. Both scenarios require network function virtualization based on solutions offering minimized image sizes to reduce the volume of virtual function repositories, and minimal transfer delays between repositories implemented in a distributed infrastructure. The optimized virtual function launch time is also significant. Security aspects in strictly isolated network slices need to be considered in the context of virtual functions and the potential exigency of installing customized firewalls at key 5G network interfaces. In response to these challenges UNFs have been proposed as a solution for 5G and next-generation cloud-based network implementations.

### Introduction to unikernels

Unikernels are specialized, single-address space machine images constructed by using library operating systems^17^. They are created by compiling the application code together with a minimal set of kernel libraries needed to run the application on specific hardware. This approach is similar to the modular architecture of the Linux kernel. However, only the necessary libraries are used in the unikernel compilation process, which greatly reduces the size of the image. Moreover, the resulting image contains a single application and lacks the usual features of a traditional operating system, such as the shell, POSIX (*Portable Operating System Interface*) utilities, etc. Because each application is different, the resulting image is always unique, hence the name unikernel.

**Figure 1:**
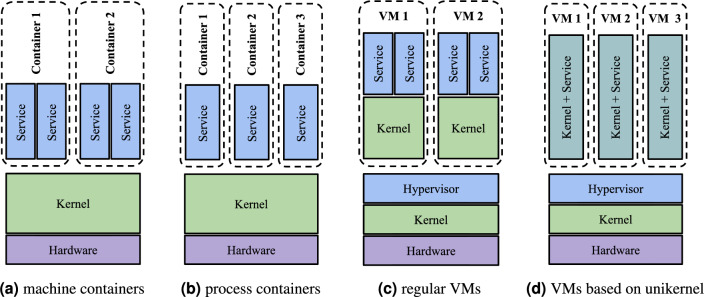
Resource isolation methods. Source^8^.

This concept is illustrated in Fig. 1. There are two basic technologies for resource isolation in the operating system: VMs and containers. While VMs require a hypervisor for the virtual hardware layer abstraction, containers run directly on top of the kernel. Although the hypervisor provides better resource isolation and thus a higher level of security, it introduces an additional overhead on the running workloads. Therefore, the performance of VMs is inferior to that achieved by containers. Another difference is the minimization and specialization of the image. While traditional VMs and machine containers can run multiple applications at the same time, process containers can run a single application only, as is the case with unikernels. Thus, unikernels resemble the idea of process containers in the hypervisor world.

Unikernel images are smaller than images of regular operating systems. As a result, they are less of a burden on machine resources, helping achieve performance results comparable to containers. They are also more secure than containers because they rely on a hypervisor to provide resource isolation. Moreover, since unikernels usually do not include standard operating system tools such as network diagnostic and inspection tools, even a compromised unikernel does not pose a threat to other hosts on the network. IncludeOS^18^ is a popular unikernel project used in research. UKL^19^, which provides a framework for building unikernels based on the Linux kernel, is one of the more recent and promising projects marking the ongoing development of this technology.

### Unikernel network functions

Since unikernels are specialized images, they are suitable for the implementation of specialized services such as network services. Let us take firewall services as an example. A typical firewall service consists of a packet inspection application code running in the user space and libraries responsible for interacting with the Network Interface Card (*NIC*) running in the kernel space. Therefore, implementing network services based on traditional operating system images introduces unnecessary overheads, which usually translates into diminished performance of a given service, which is critical in the case of network services. Therefore, implementing network services based on unikernels makes much more sense.

Moreover, since unikernels are almost as lightweight as containers, TSPs can use them for cloud-native network service implementation. In such a situation, the network service is broken down into smaller components, known as network functions, which perform certain individual operations and communicate with each other. The following types of network functions are known: Physical Network Functions (*PNFs*), Virtual Network Functions (*VNFs*) and CNFs. Unikernel Network Functions, or UNFs, are simply a new type of network function which uses unikernels for service implementation. Such network functions benefit from all the advantages of a hypervisor while achieving comparable performance results to CNFs^20^.

**Figure 2:**
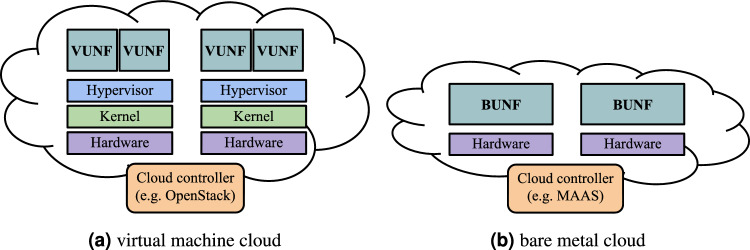
UNFs use cases.

However, UNFs do not necessarily require a hypervisor. Since unikernels are machine images, they can also run directly on bare metal machines. The two use cases are shown in Fig. 2. The first type of UNFs uses a hypervisor and runs inside VMs. We call them Virtual UNFs (*VUNFs*). Due to their small size and the ability to run several network services on a single bare metal machine, they are applicable to virtual Customer Premises Equipment (*vCPE*)^21^ and mobile edge^22^ NFV use cases. OpenStack can be used as a Virtual Infrastructure Manager (*VIM*) in this case. The second type of VNFs runs directly on bare metal machines. Such UNFs, known as Bare-Metal UNFs (*BUNFs*), can use Metal-as-a-Service (*MAAS*) as VIM^23^ and, due to their improved performance, are suitable for the 5G core and vEPC NFV use cases.

## Performance evaluation

In order to evaluate the performance of VUNFs, the following experiment was carried out. Benchmarking methodology for firewall service^24^ was used to examine how various performance parameters of a firewall service change depending on the number of firewall rules. The firewall service was implemented in four various technologies—KVM, Docker, LXD and IncludeOS—allowing us to compare the results achieved by unikernels with results achieved by other technologies.

The firewall service for KVM, Docker and LXD technologies was implemented based on the Ubuntu 18.04 image and the iptables software. This is because Ubuntu is the most popular cloud image and iptables is the most popular firewall software for Linux. In turn, the firewall service for IncludeOS was implemented using the IncludeOS NaCl interface^25^. Summarizing, the unikernel was executed as a virtual machine on top of the KVM hypervisor and the containers were run on bare metal. It is worth mentioning the rules were exactly the same in both cases and offered the same functionality. The lab environment consisted of three physical hosts—Sender, Firewall and Receiver—and a dedicated 1 Gb/s network which was used to connect the hosts in a chain. The firewall service was run on the top of the Firewall host using either virtualization or container technologies. The number of rules in a TSP firewall depends on the security policy. Therefore, we conducted large-scale tests to check the performance in various scenarios. In each experiment various images with the number of firewall rules of 1, 10, 100, 1000, 10,000, 100,000 and 1,000,000 were used.

The following subsections contain the experimental results. Each one describes a dedicated experiment which was carried out to measure different performance parameters of the firewall service. In each case, the results are presented as a table and chart. Each graph uses a logarithmic scale on the *x* axis.

### Image size

**Figure 3:**
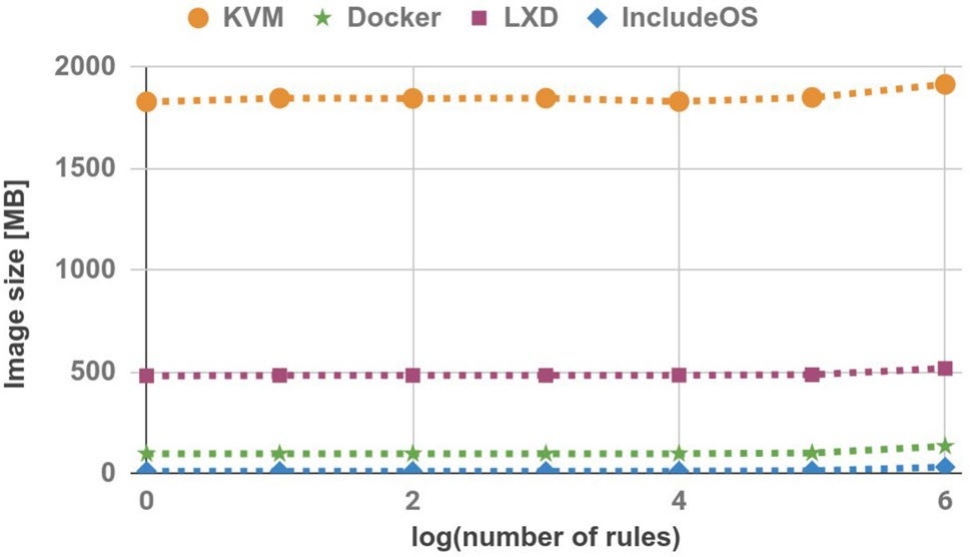
Image size.

The experiment started by comparing the image sizes of all images used in the experiment. The image sizes were measured using standard Linux tools and are shown in Table 1 and Fig. 3. The IncludeOS images are smaller than other images. Moreover, the size of IncludeOS images increases more slowly in comparison with the other technologies, as the number of firewall rules increases.

**Table 1:** Image size (MB).

No. of rules	KVM	Docker	LXD	IncludeOS
1	1829.5	95.9	480	9.3
10	1847.5	95.9	482	9.3
100	1846.2	95.9	482	9.3
1000	1847.5	96	482	9.3
10,000	1831.1	96.3	483	9.5
100,000	1851	99.5	486	12
1,000,000	1915.7	133	517	30

### Launch time

**Figure 4:**
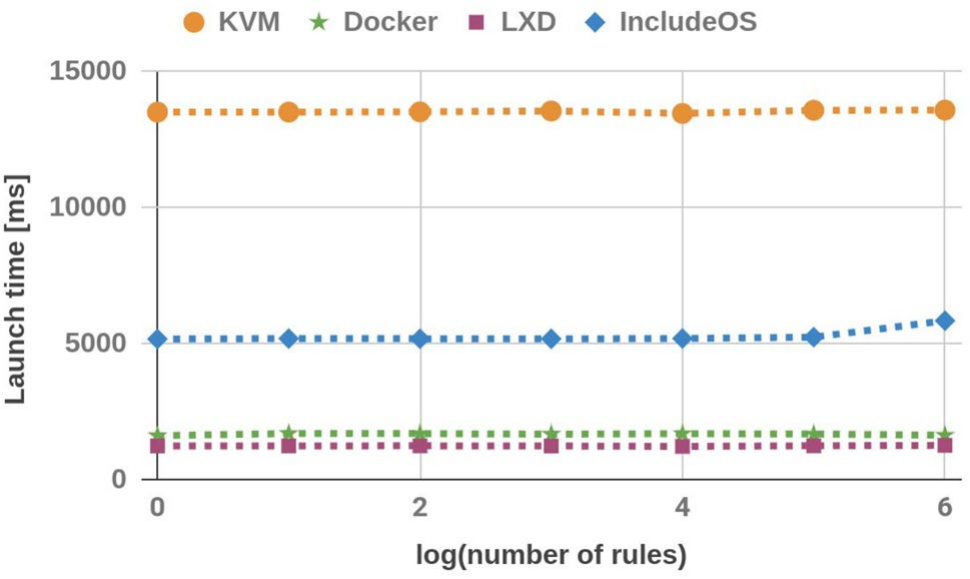
Launch time.

The launch time of all images was measured next. In order to achieve this, we wrote a script which measures the time between image initialization and the first response to the Internet Control Message Protocol (*ICMP*) echo request sent from the Firewall host to the image. The launch time was measured 100 times to generate the average value. The average values are shown in Table 2 and Fig. 4. Although the launch time of IncludeOS images is longer than the launch time of containers, it is still shorter than KVM Ubuntu images. Additionally, the launch time of the IncludeOS image of the firewall with 1,000,000 rules is significantly longer than the launch time of other IncludeOS images.

**Table 2:** Launch time (ms).

No. of rules	KVM	Docker	LXD	IncludeOS
1	13,515	1610	1234	5168
10	13,516	1691	1236	5185
100	13,527	1687	1238	5179
1000	13,557	1666	1232	5173
10,000	13,467	1685	1216	5186
100,000	13,583	1672	1240	5236
1,000,000	13,590	1627	1255	5840

### Idle ping delay

**Figure 5:**
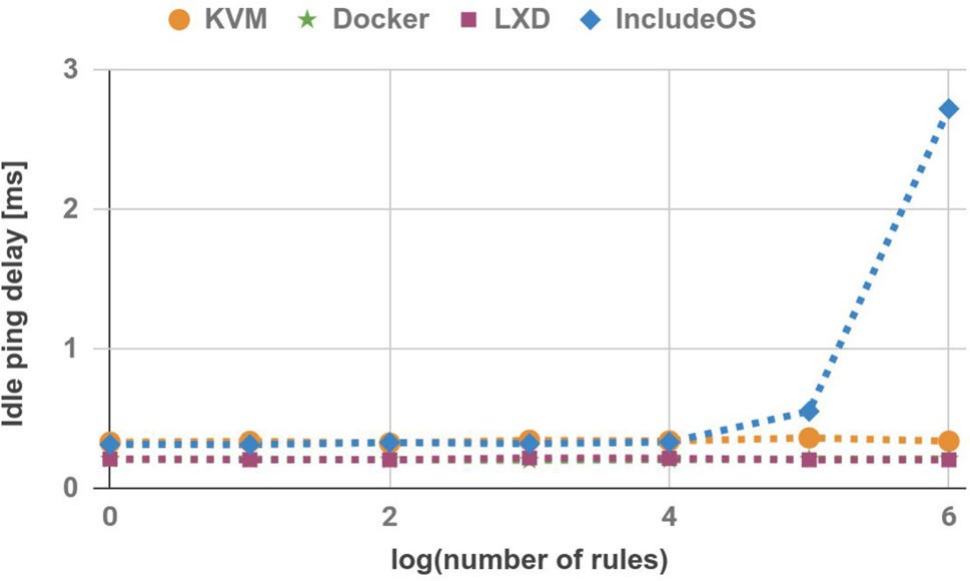
Idle ping delay.

In the next step, we measured idle ping delay by sending 100 ICMP echo requests from the Sender host to the image and generating the average. Ping probe was sent every second which is a regular ping time interval. The results are shown in Table 3 and Fig. 5. For images with a low number of rules, IncludeOS images achieve worse results than containers, although they are comparable to KVM Ubuntu images. However, once firewall images reach 100,000 rules, the idle ping delay for IncludeOS increases rapidly. Probably, the NaCl implementation in the IncludeOS project was not ready for use cases with the largest number of rules.

**Table 3:** Idle ping delay (ms).

No. of rules	KVM	Docker	LXD	IncludeOS
1	0.33	0.208	0.209	0.314
10	0.336	0.208	0.205	0.313
100	0.324	0.204	0.204	0.328
1000	0.344	0.201	0.217	0.32
10,000	0.339	0.205	0.215	0.331
100,000	0.361	0.209	0.204	0.552
1,000,000	0.338	0.21	0.204	2.724

### TCP throughput

**Figure 6:**
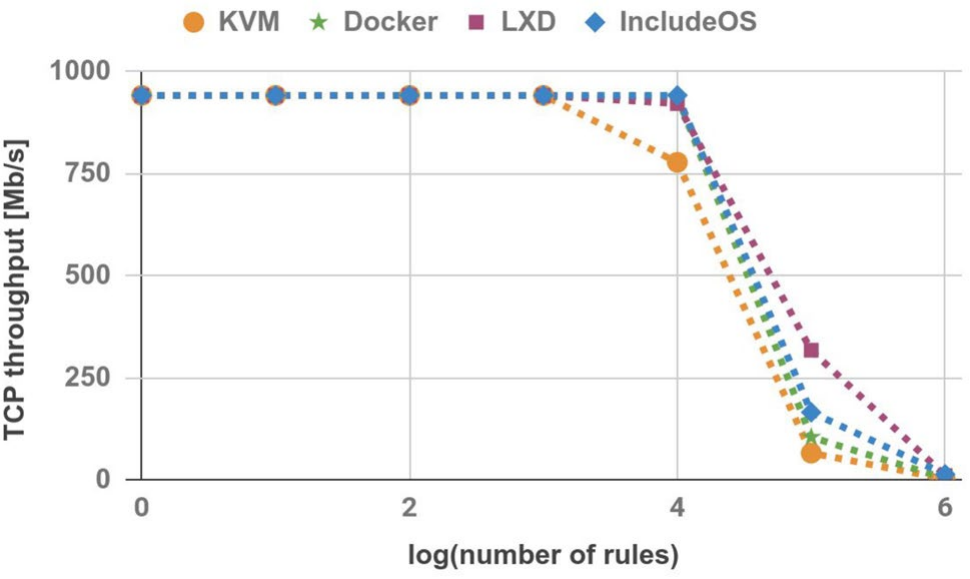
TCP throughput.

TCP throughput was measured by initiating an iperf^26^ session between the Sender and the Receiver hosts. Each session lasted 30 seconds. The results are shown in Table 4 and Fig. 6. The only case where IncludeOS achieves worse results than other technologies is a firewall image with 100,000 rules. In this case, the TCP throughput of the LXD-based firewall is almost double.

**Table 4:** TCP throughput (Mb/s).

No. of rules	KVM	Docker	LXD	IncludeOS
1	942	942	942	942
10	942	942	942	942
100	942	942	942	942
1000	942	942	942	942
10,000	778	939	922	942
100,000	65	104	317	165
1,000,000	2	3	10	13

### UDP throughput

**Figure 7:**
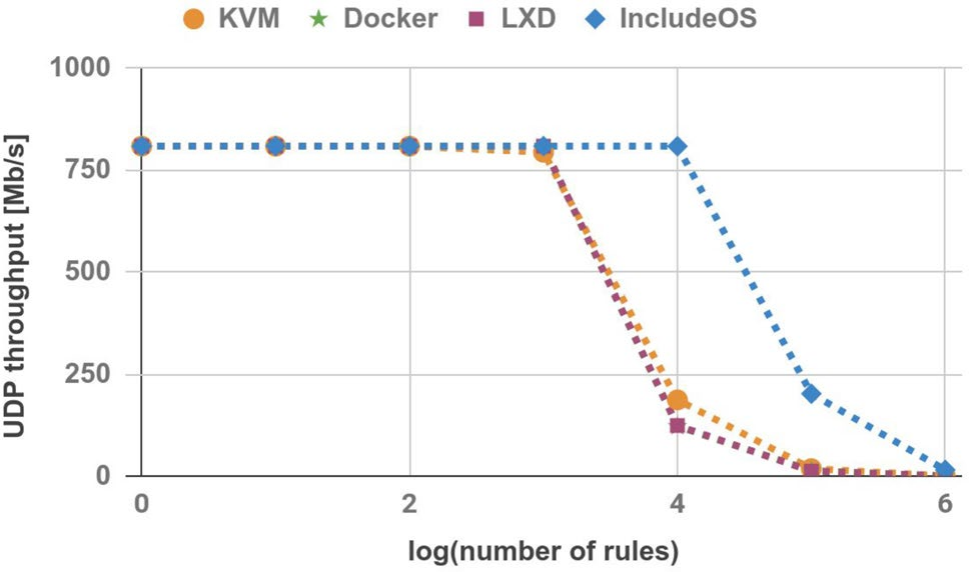
UDP throughput.

The TCP throughput experiment was followed by UDP throughput measurements, once again using iperf. Another session between the Sender and the Receiver hosts was established. This session also lasted 30 s. The results of this experiment are shown in Table 5 and Fig. 7. In this case, the results achieved with IncludeOS are vastly superior, in line with the results presented in^9^. Regardless of the number of rules, the UDP throughput of IncludeOS images is higher compared to other technologies.

**Table 5:** UDP throughput (Mb/s).

No. of rules	KVM	Docker	LXD	IncludeOS
1	809	809	809	809
10	809	809	809	809
100	809	809	809	809
1000	794	809	809	809
10,000	187	122	124	809
100,000	18	13	12	202
1,000,000	0	0	0	15

### TCP requests per second

**Figure 8:**
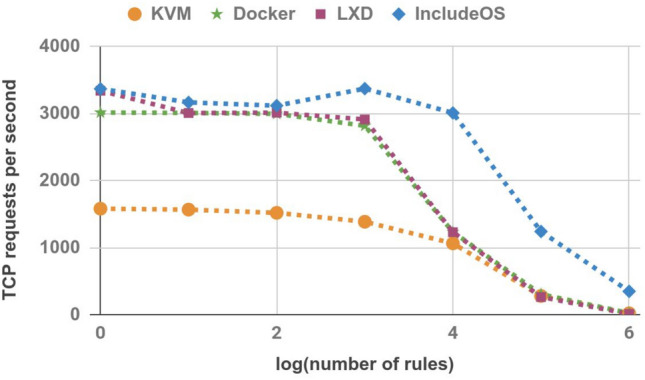
TCP requests per second.

In the next experiment, we measured TCP requests per second. A netperf^27^ session was established between the Sender and the Receiver hosts. As in previous experiments, the session lasted 30 s. The results are shown in Table 6 and Fig. 8. This is another case where IncludeOS achieves better results than any other technology, regardless of the number of firewall rules.

**Table 6:** TCP requests per second.

No. of rules	KVM	Docker	LXD	IncludeOS
1	1581	3016	3342	3371
10	1567	3011	3009	3168
100	1519	2997	3014	3119
1000	1386	2819	2914	3374
10,000	1063	1233	1231	3011
100,000	278	305	263	1241
1,000,000	19	22	9	347

### UDP requests per second

**Figure 9:**
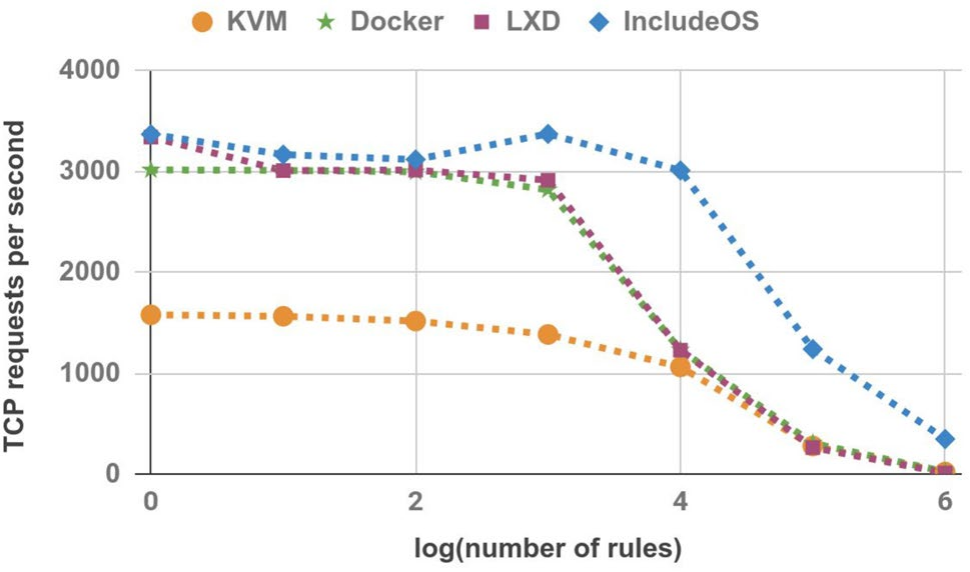
UDP requests per second.

Netperf was also used to measure UDP requests per second. Another session was established between the Sender and the Receiver hosts, once again running for 30 s. All results are shown in Table 7 and Fig. 9. Although IncludeOS achieves worse results than Docker for a low number of rules, once the firewall image reaches 10,000 rules the situation changes and IncludeOS performs significantly better than the other technologies.

**Table 7:** UDP requests per second.

No. of rules	KVM	Docker	LXD	IncludeOS
1	1758	3399	3354	3214
10	1701	3400	3042	3231
100	1586	3395	3012	3201
1,000	1419	3405	2843	3191
10,000	1006	1223	1231	3182
100,000	278	292	274	1280
1,000,000	19	16	15	379

### TCP connections per second

**Figure 10:**
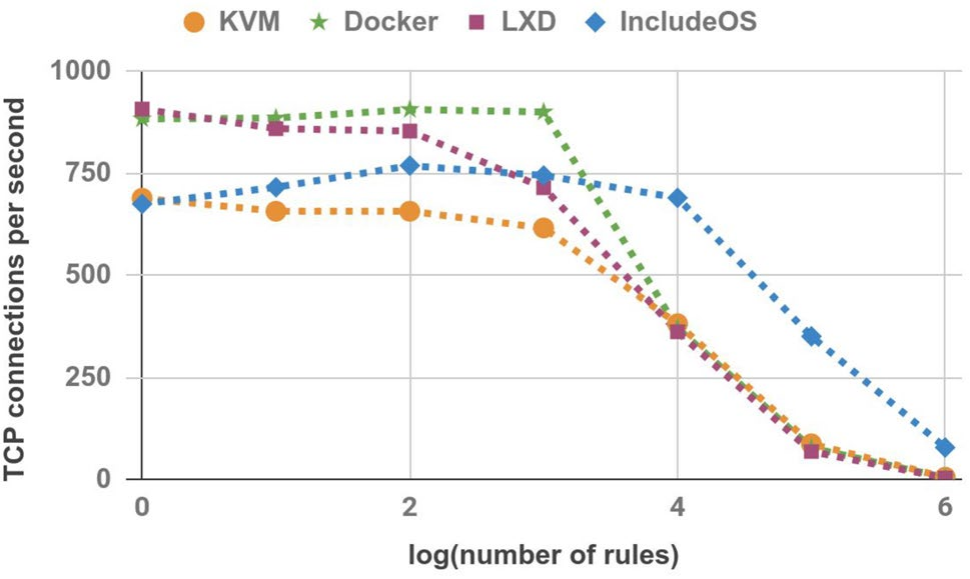
TCP connections per second.

The previous experiments were followed by measurements of TCP connections per second. Netperf was used once again. We established another session between the Sender and the Receiver hosts, once again running for 30 s. The results are shown in Table 8 and Fig. 10. Although for firewall images with a low number of rules IncludeOS achieves worse results than container technologies and even KVM Ubuntu images, once the firewall image reaches 10,000 rules it can handle more TCP connections per second than other technologies used in the experiment.

**Table 8:** TCP connections per second.

No. of rules	KVM	Docker	LXD	IncludeOS
1	689	884	908	675
10	657	886	860	716
100	657	907	854	769
1000	616	901	715	745
10,000	382	371	362	690
100,000	87	77	68	351
1,000,000	5	4	3	78

### ICMP latency

**Figure 11:**
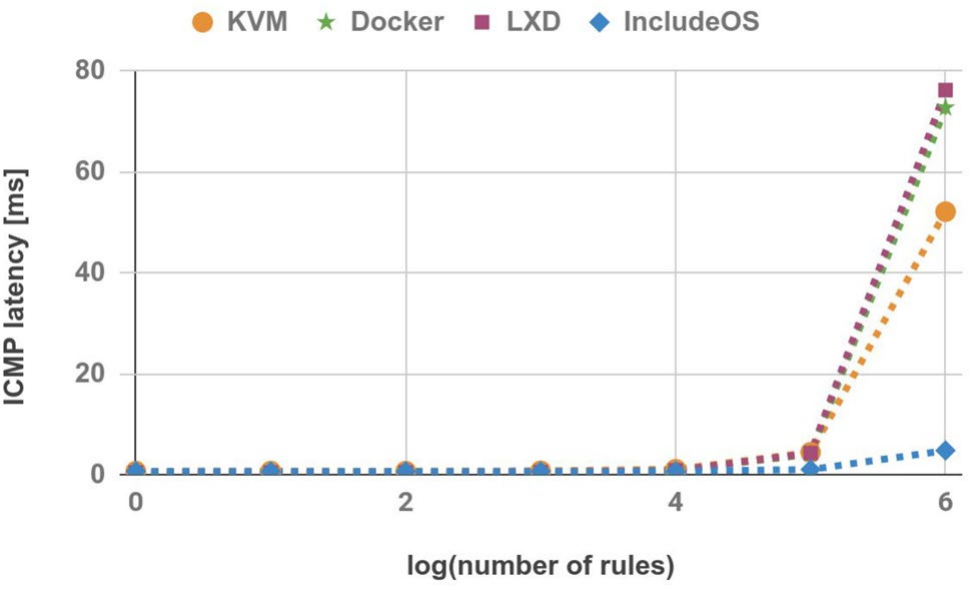
ICMP latency.

In the last two experiments, we measured the latency of the traffic flowing between the Sender and the Receiver hosts. In order to measure the ICMP latency, 100 ICMP echo requests were sent from the Sender to the Receiver to obtain the average. The average values are shown in Table 9 and Fig. 11. Although the ICMP latency of IncludeOS images is higher than the ICMP latency of containers for firewall images with a small number of rules, once the firewall image reaches 10,000 rules the latency is the lowest. Moreover, for an image with 1,000,000 rules the ICMP latency for other technologies increases rapidly, while for IncludeOS the rate is more stable.

**Table 9:** ICMP latency (ms).

No. of rules	KVM	Docker	LXD	IncludeOS
1	0.686	0.429	0.428	0.635
10	0.697	0.427	0.43	0.619
100	0.682	0.437	0.436	0.619
1000	0.747	0.422	0.494	0.62
10,000	1.067	0.845	0.824	0.652
100,000	4.428	4.035	4.257	1.004
1,000,000	52.14	72.784	76.233	4.774

### TCP latency

**Figure 12:**
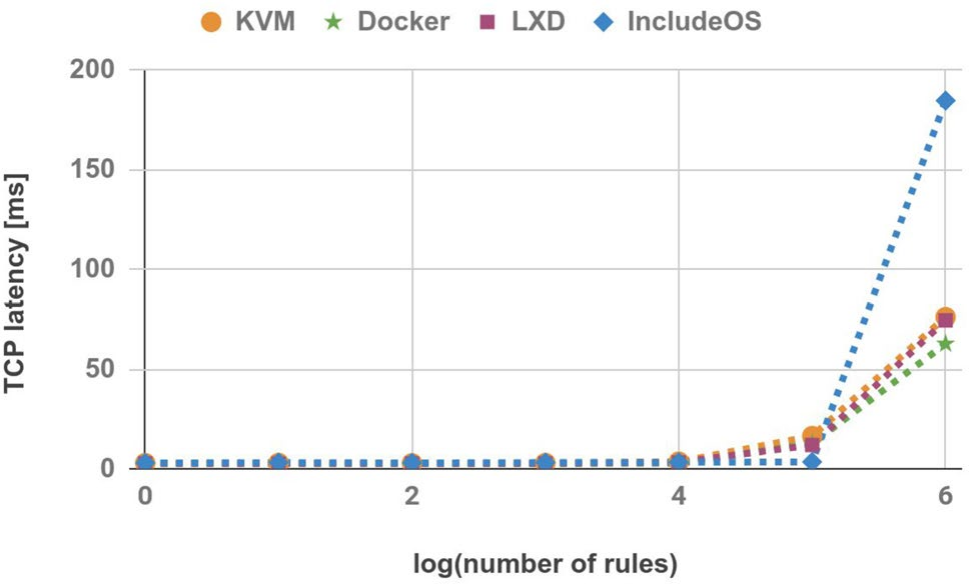
TCP latency.

The final experiment involved measuring TCP latency using the hping3 tool^28^. One hundred TCP packets were sent from the Sender to the Receiver and the average TCP latency was derived. The average values are shown in Table 10 and Fig. 12. In this case IncludeOS achieves worse results than other technologies used in the experiment. The only exception is an image with 100,000 rules where the TCP latency for other images is already increasing, while it remains stable for IncludeOS.

**Table 10:** TCP latency (ms).

No. of rules	KVM	Docker	LXD	IncludeOS
1	2.826	2.76	2.619	2.901
10	2.91	2.695	2.583	3.139
100	2.745	2.683	2.596	2.951
1000	2.89	2.652	2.668	3.132
10,000	3.554	2.769	3.191	3.206
100,000	16.435	12.688	11.954	3.441
1,000,000	76.2	62.8	74.6	184.755

## Observations and discussion

By conducting an extensive investigation of various parameters of a firewall service implemented in four different technologies, we evaluated the performance of unikernels and VUNFs and compared them to traditional VMs and containers. The data presented in the previous section shows that IncludeOS images achieve inconclusive results and the exact numbers vary as the number of firewall rules changes. However, the following observations can be made.

First of all, in most cases IncludeOS images achieve better performance results than KVM Ubuntu images. This is understandable, since IncludeOS images are more lightweight compared to Ubuntu images, while underneath they use the same virtualization technology of KVM. Moreover, in some cases IncludeOS images achieve better results than container technologies. These parameters include image size, UDP throughput, TCP requests per second and ICMP latency. Finally, IncludeOS usually deals better with firewall services with greater numbers of rules.

## Limitations and future work

Although the experimental results presented in the previous sections provide a solid overview of the performance of the investigated technologies, it is important to mention some limitations of the method used in this paper. While firewall images for KVM, Docker and LXD were prepared based on the Ubuntu image and iptables software, IncludeOS images were compiled using the IncludeOS NaCl interface. This means that the actual firewall implementation was different for IncludeOS and other technologies. It should be noted that although we attempted to use the iptables source code to compile IncludeOS images, this was unsuccessful due to the limitations of IncludeOS itself. Other limitations of IncludeOS, such as high RAM consumption and long compilation time when compiling firewall images with a high number of rules, are mentioned in^8^. It should also be noted that the research carried out did not cover a bare metal implementation. This was due to the limitations of the testing environment itself—this option was not available in the IncludeOS project at the time of writing this article. The only available option was the KVM build. There were also some limitations imposed by the tools used for data collection (e.g., a lack of standard deviation of the measurements). We used typical, widely used engineering tools, utilizing their regular methods and settings for collecting and presenting measurement data. Consequently, in the case of ICMP delay, TCP delay, TCP/UDP throughput, TCP connections per second, the results are presented only in a manner typical for the applied tools such as iperf, netperf, hping3.

Future work should investigate the performance of a custom network service when implemented in all four technologies. Such a service would run as a process inside the KVM, Docker and LXD images. Its code would be used directly when compiling IncludeOS images instead of using the NaCl interface. This means that the service would need to be implemented in C. A relatively simple example of such a service would be a packet scanner. Potential future research may also include a study on the implementation of a variable number of virtual machines and assessing its impact on system performance. Work in this area would be both purposeful and interesting. The performance evaluation of Unikernels is particularly valuable and interesting when comparing the computing resource requirements, such as CPU and RAM usage, of the examined systems. A comparative analysis of this kind would be significant for a meaningful assessment of the considered solutions.

## Conclusions

In response to the challenges related to network function softwarization, such as increasing demand for improved performance and security, TSPs have started exploring alternatives to traditional virtualization. Unikernels are one such technology, making it possible to compile specialized, embedded, highly secure images. Due to their lightweight nature they are also expected to achieve performance results comparable to containers. Network functions based on unikernels, referred to as UNFs, can either run as VMs (VUNFs) or directly on bare metal machines (BUNFs).

This paper presents an evaluation of VUNFs. For this purpose a firewall service was implemented in four different technologies—KVM, Docker, LXD and IncludeOS—and basic performance parameters were investigated through a set of experiments and benchmarks based on official benchmarking methodology for firewall performance. Analysis of the results of the experiments revealed that IncludeOS generally achieves better performance results than KVM images based on Ubuntu and iptables. In certain cases, such as UDP throughput, TCP requests per second and ICMP latency, IncludeOS also achieves better results than containers. Finally, we found that IncludeOS generally deals better with firewall services with a high number of rules.

## Data Availability

The authors declare that data supporting the findings of this study (performance evaluation) are available within the article.
